# Old-age insecurity: How far does the “Parents and Senior Citizen’s Act of 2007” address the problem?

**DOI:** 10.4103/0019-5545.74302

**Published:** 2010

**Authors:** P. K. Kuruvilla

**Affiliations:** Department of Psychiatry, St. Gregorios Mission Hospital, Parumala - 689 626, Pathanamthitta District, Kerala, India

As is well known, old age is seen to be a time of losses - declining health, failing sensory inputs and shrinking networks. In India, as opposed to the wealthy nations, there is a paucity of salaried jobs and social security. Although India gained her independence six decades ago, there have not been any tangible measures to address the financial insecurities of the aged save for well-meaning directives in the constitution and announcements of utopian schemes in the parliament from time-to-time. The Act of 2007 is a measure which addresses directly the issue of ownership of property by the aged and the financial upkeep by their relatives; it is worthwhile, in fact edifying, to go through the laws in this regard, both before the 2007 Act and in which way the Act differs.

## THE EXTENT OF THE PROBLEM

Yes, demographers have been cautioning us about the graying of our populations for quite some time now. The proportion of the elderly, formally defined as those above 60 years has been increasing the world over. The elderly population in India accounted for 5.6% of the total population in 1971. As per the 1981 census, 6.3% of Indians were above 60 years. In 2007, the figure hovered around 7.5%, and this is predicted to be 12.5% or about one out of eight by 2025. In absolute numbers, the census of 2001 revealed that there were 77 million elderly whereas in 1991, their count was only 57 million. During 1991-2001, the growth rate among the 60+, 70+ and 80+ was much higher than the general population growth rate of 2% per annum. Another pertinent statistic, in fact a more refined measure, is the ‘Old Age Dependency Ratio’. This is the number of those above 65 divided by those between 15 and 60 or roughly the number of old, divided by the number in the work force. In 2001 it was 11.9% and it is projected to be 28.2% by 2050 for the whole of India. Other aspects of the problem will be that this will be more so in the rural areas, and that there are more women elderly than men.[1] The ratio of women to men is 1028 per 1000. This is a reversal of the ratios in the young. The chances of widowhood are high, given the fact that women have higher survival rates and that they tend to marry men who are much older than them. According to census figures of 2001, and National Sample Survey Organization data,[2] 63% of males and 58% of females work beyond 60 years, perhaps mostly out of economic compulsions. Compare this to only 2% of those above 65 who work in some of the developed countries.[3] A sample survey in rural India reflected a greater degree of insecurity among the aged. This is more so in women.[4] In this context, it appears that ownership of properties and assets could well be a defining condition of aged peoples’ security. In the country as a whole, the basic needs of half the elderly are not met with.[5] Since this leads to insecurity, and dependency is an important dimension of their well-being[6,7] rectifying the situation will, to a measure, improve the well-being.

## HISTORY

The chapter IV of the constitution (coming under the Directive Principles) has taken up the concerns of the elderly. Nonetheless, these are not enforceable by the law. In 1956, after Independence, the Hindu law made a statutory provision for the elderly. Maintenance of parents by their children (sons and daughters) is subsumed in section 20 of the Hindu Adoption and Maintenance Act of 1956. A similar clause for sons to look after their parents existed under the Muslim Law, too. However, here too it remained only a moral obligation. In 1973, for the first time, a provision was introduced in the code of criminal procedure. An elderly person could thus move court for his maintenance. It is, however, essential that the other party has sufficient means and has neglected or refused to maintain the parent who is unable to take care of himself. This law is secular and includes married daughters in addition to sons. However, the parent needs to approach the court against an offspring, and the onus was on the parent to prove that the other party has sufficient means and has refused to maintain him i.e., the parent. This is harsh and cumbersome as well. Even at the time of its inception, the Law Commission was not in favor of making such a provision. It remarked that, “The Criminal Procedure Code is not the proper place for such a provision. There will be considerable difficulty in the amount of maintenance awarded to parents amongst the children in a summary proceeding of this type. It is desirable to leave this matter for adjudication by the Civil Courts.”

## THE ACT OF 2007

The Indian parliament has passed in 2007 the Act the “Parents and Senior Citizen’s Act of 2007.”[8] This will come into force in a state once the concerned state enforces it. The date on which the Act comes into effect will be notified by the concerned state government in its official gazette. This bill seeks to make it a legal obligation for children (including grand children) and heirs to provide maintenance to senior citizens. It includes in its purview all Indian citizens, including those living abroad. The state government may set up maintenance tribunals, one or more in every subdivision, to decide the level of maintenance. Appellate tribunals are to be set up at the district level. In this manner, the filing of an appeal is simplified and there is a directive to determine the level of maintenance within 90 days. Any maintenance order made by the tribunal shall have the same force as an order passed under Chapter IX of the code of criminal procedure. If the tribunal is satisfied that there is neglect, it may order the children/relative to give a monthly allowance of up to Rs10000. Unlike moving court, the procedure for filing petitions to the tribunal is seen to be simpler. Moreover, a voluntary organization registered with the government can do this for an elderly person, as also another person on behalf of a senior citizen. The tribunal is also given the authority to file a petition ‘suo moto’. In case of childless persons, the relatives are obliged to look after them. The bill defines the term ‘relative’ as someone who is in possession of or would inherit a senior citizen’s property.

On failure to comply with the maintenance, the tribunal may issue a warrant within 3 months of the due date. Should it remain unpaid, the accused may be imprisoned up to 1 month/till the time it is paid – whichever is earlier. Punishment for abandoning a senior citizen shall include imprisonment for up to 3 months or a levy of rupees 5000/ or both. The tribunal can declare a transfer of property (as gift or otherwise) from a senior citizen to a transferee as void if the transfer was made under the condition of maintenance and the transferee neglects the agreement. Only parents are allowed to appeal against the tribunal.

## OTHER RELEVANT MEASURES PASSED IN THE PARLIAMENT IN 2007

In this context, it appears relevant to add on about two new schemes, announced in the budget speech in the year 2007.

Reverse Mortgage: The 2007 Budget Speech announced the introduction reverse mortgage for senior citizens by the National Housing Board. The scheme involves the senior citizen borrower mortgaging the housing property to a lender, who then makes periodic a payment to the borrower(s) during the latter’s life time.

New Pension Scheme: Under the NPS, every subscriber is to have an individual pension account, portable across job changes. The amount (including the income on investments) will be available at the age of 60 years, with at least 40% to be converted to monthly payments for the rest of their lives. This pension scheme wants to bring into its fold, those working in the unorganized sector, so that they can save for their old age right from the time they start earning.

## CRITICISMS AND SUGGESTIONS

The criticisms of the Act include its ambiguities.[9,10] Senior citizen is defined as “any person above 60 years and includes parent, whether or not senior citizen”. This implies that a parent with a child who is not a minor whether or not 60 years will be defined as a senior citizen. There is scope for ambiguity. “Relative” means any legal heir of the childless senior citizen who is not a minor and is in possession of or would inherit his property after death. Does that mean that the content of the person’s will should be revealed beforehand and does that also imply that there will be no changes about the inheritance of property once a will is written? The bill also proclaims that the state government should set up old-age homes in every district and that each is to house at least 150 people. Although this clause is added with the indigent elderly in mind, it is not clear as to why the bill has to specify the size of old-age homes. Some have opined that the government is side-stepping its own responsibility by not coming up with more supportive measures for the senior citizen and instead is passing on much of the responsibility to the children, grand children and heirs. A few have also raised the concern that only the better off with property and children will benefit with the bill coming in to effect. Although easier than moving the court, parents and the elderly would still have reservations about approaching a tribunal against their offspring and close relatives. The statistics obtained from the honorable High Court of Kerala reveal that since the Act was enforced in September 2008, i.e., roughly 2 years, the total number of cases filed has been 746, out of which 178 cases have been decided upon. For a state with 30 million population this is a modest number. The National Commission for Women which was constituted in 1990 seems a good model to follow. Here there is more emphasis on reaching out and in generating general awareness among women, and also assisting women in redressing their grievances. The Women’s Commission also undertakes the review of existing provisions in the constitution, and has the mandate to take up promotional and educational research. Finally, since the police force will have to actually enforce the decisions of the tribunals, their performance in this regard has to be evaluated from time-to-time.

Notwithstanding these loop holes and lacunae, the 2007 Act and the new schemes for the elderly can be seen as significant steps in erasing some of the insecurity of the elderly and thereby improving their sense of well being.
